# Patient-specific factors modulate leukocyte response in dimethyl fumarate treated MS patients

**DOI:** 10.1371/journal.pone.0228617

**Published:** 2020-02-11

**Authors:** Myla D. Goldman, Lauren Dwyer, Rachael Coleman, Min-Woong Sohn, Olaf Stuve

**Affiliations:** 1 Virginia Commonwealth University School of Medicine, Department of Neurology, Richmond, Virginia, United States of America; 2 Hendrix College, Conway, Arkansas, United States of America; 3 Department of Public Health Sciences, University of Virginia, Charlottesville, Virginia, United States of America; 4 University of Texas Southwestern Medical Center at Dallas, Department of Neurology and Neurotherapeutics, Dallas, Texas, United States of America; University of Iowa, UNITED STATES

## Abstract

**Objective:**

Determine if patient-specific factors modulate absolute lymphocyte count (ALC), neutrophil count (ANC), and/or Neutrophile-lymphocyte ratio (NLR) in Dimethyl Fumarate (DMF) treated patients.

**Methods:**

A retrospective study of patients who initiated DMF between 2013–2018. A multicenter study of two MS clinics: Charlottesville, VA (UVA) and Dallas, TX (DaVA)

**Results:**

103 patients (67-UVA, 36-DaVA) met eligibility. At baseline, the DaVa population was younger (mean±sd: 38.6±9.0 vs 42.2±12.5, p 0.152) and had a higher proportion of males (61% vs. 35%), consistent with a veteran cohort. Pre-treatment, all other laboratory parameters were similar between the two groups. On treatment there was a 30% lowering of mean ALC, with 3% having grade-3 lymphopenia (ALC < 500). Sustained neutropenia occurred in 3.9% of patients and was more common in males. Over 50% of patients had a high NLR at baseline, with a further 44% increase in NLR on-treatment. Age was significantly predictive of lymphopenia, with grade-3 lymphopenia found in 33% of patients ≥ 55 years. Neutropenia was more common in males. Serum BG (sBG) has modest correlation to leukocyte parameters. BMI was not correlated with any leukocyte-related outcomes.

**Conclusions:**

Patient-specific factors, specifically–age, sex, and serum blood glucose, modulate leukocyte response and ratios in DMF treated MS patients. Age appears to be a relevant predictor of lymphopenia and should be a factor in treatment decision making. Neutropenia, independent of lymphopenia, can occur and males may be at increased risk. High sBG may impact leukocyte count and ratios in MS patients and merits further study, particularly in patients with diabetes. NLR is abnormal in MS and increased with DMF-treatment, the clinical implications of this will require further study.

## Introduction

Dimethyl fumarate ((DMF), *Tecfidera*, Cambridge MA) is an oral agent approved in March 2013 for treatment of relapse-remitting multiple sclerosis (RRMS). In phase 3 clinical trials, approximately 4–5% of patients developed grade 3 lymphopenia classified as an absolute lymphocyte count (ALC) less than 500 [1,2]. A more recent study found that 6% of DMF treated patients at a clinic developed grade 3 lymphopenia [3], with 20% of the patients over age 55 developing grade 3 lymphopenia [4]. Lymphopenia appears to be a risk factor for the rare complication of progressive multifocal leukoencephalopathy (PML). To date, seven DMF-associated cases of PML have been reported, three of which occurred in patients with grade 3 lymphopenia and a fourth in the setting of grade 2 lymphopenia (ALC of 500–799) [5]. Despite the recognized risk of lymphopenia with DMF, there is limited understanding of which patients may be most at risk to develop lymphopenia.

While DMF generated lymphopenia has been analyzed by multiple studies, DMF effects on neutrophils have mostly been studied in animal models. Experimental autoimmune encephalomyelitis animal models have suggested that neutrophils contribute to the demyelination process [6]. Using mice models, DMF has been proven to be an agonist to the hydroxycarboxylic acid receptor 2 (HCA2) which hinders neutrophil migration to the central nervous system [7]. In animal models and a small clinical study, peripheral blood neutrophil count was not affected by DMF [7, 8] We examined if DMF treated MS patients in a larger clinical study experienced neutropenia and determined if any factors could predict the absolute neutrophil count (ANC) of patients.

Beyond the absolute counts of neutrophils and lymphocytes, the neutrophil-lymphocyte ratio (NLR) may be an important metric. The NLR appears to be a prognostically meaningful outcome in several disorders, including the metabolic syndrome [9], Obesity [10], and diabetes [11]. Recently, the neutrophil-to-lymphocyte ration was proposed as a disease marker in MS [12]. Our previous work has focused on the importance of glucose regulation in MS outcomes [13]. Interestingly, a recent case report detailed a potential role of DMF in glucose dysregulation [14].

## Methods

### Standard protocol approvals, registrations, and patient consents

This retrospective chart review study was approved by the Institutional Review Board at the University of Virginia with a waiver of consent approved by the IRB. The study was executed in accordance with the Health Insurance Portability and Accountability Act of 1996.

### Study population and procedures

We performed a retrospective database search to identify DMF treated MS patients at the University of Virginia James Q. Miller MS clinic (UVA) and at the Veterans Affairs North Texas Health Care System in Dallas (DaVA). Patient characteristics and laboratory values were acquired from the electronic medical records of both population centers for patients treated between March of 2013 and May of 2018 with the following eligibility criteria: over age 18, confirmed diagnosis of MS (per McDonald criteria [15]), and DMF treatment. Patients with diabetes or taking glucose regulating medications were excluded. Laboratory draws within one week of steroid administration were excluded. Data collected from the electronic medical records of both population centers included age, sex, body mass index (BMI), DMF treatment start date, date of laboratory draws, sBG, white blood cell count (WBC), ALC, and ANC. Lymphopenia was classified as mild = ALC > 800 (grade 1) and severe = ALC of <500 (grade 3). Neutropenia was defined as ANC < 1.5. The NLR was calculated using the ratio between the ALC and ANC. NLR values > 2 were considered elevated for analysis. High serum BG values were defined as those greater than 2 standard deviations above the mean (sBG > 180). Baseline laboratory values were collected within 12-months prior to DMF start and on-treatment laboratory values were collected at non-standard intervals as clinically indicated.

### Statistical analysis

We compared patient demographic characteristics between the two population centers using two-sample t-tests. Pearson’s Correlation Coefficients were used to determine if any two of the variables were related. Mixed effect regression analysis was used to examine the association between the ALC, ANC and NLR and the other variables. Mixed model regression adjusting for repeated measures was used for predictive modeling. Two-tailed p-values of <0.05 were considered statistically significant. All analyses were completed in SAS 9.4 and Stata SE 15.0.

## Results

The retrospective database search identified 103 DMF treated patients that meet eligibility criteria at the two centers (UVA = 67, DaVA = 36) with a total of 380 on-treatment lab draws available for analysis. Table 1 gives details about the demographic characteristics for the total population and the individual centers. The combined cohort had a mean age of 41.7 years and 56% were female. At baseline, the DaVA population was younger (mean±sd: 38.6±8.9 vs 43.3±12.1, p < 0.001) and had a higher proportion of males (61% vs 35%), consistent with a veteran population. The study sample included both treatment niave and DMT switchers. However, all patients had normal leukocyte counts at pre-DMF baseline. Pre-treatment, all other lab parameters were similar between the two groups. There was an increase in mean serum BG values on-treatment relative to baseline [baseline mean (sd) sBG: 98.5 (25.0); on-treatment mean (sd) sBG: 103.6 (34.9)] (Table 1).

**Table 1:** Subject demographics and serum blood glucose levels and leukocyte counts*.

Variables	Combined			DaVA			UVA			P-Value
	N	mean (SD)		N	mean (SD)		N	mean (SD)		
All	103	100.0%		36	100.0%		67	100.0%		
% Female	57	55.3%		14	38.9%		43	64.2%		**0.014**
Baseline										
Age	80	40.6	(11.1)	36	38.6	(9.0)	44	42.2	(12.5)	0.152
BMI	80	28.8	(6.6)	36	29.2	(6.0)	44	28.5	(7.1)	0.614
sBG	74	98.5	(25.0)	36	99.7	(23.0)	38	97.3	(27.0)	0.685
WBC	78	6.7	(2.0)	36	6.5	(2.1)	42	6.9	(1.9)	0.389
ALC	69	1.9	(0.8)	36	2	(0.8)	33	1.9	(0.7)	0.932
ANC	69	3.9	(1.6)	36	3.7	(1.7)	33	4.2	(1.4)	0.138
NLR	69	2.5	(1.9)	36	2.4	(1.9)	33	2.7	(1.8)	0.488
On Treatment										
Age	413	43.9	(11.1)	101	40.4	(9.7)	312	45	(11.3)	**< 0.001**
BMI (Most recent BMI)	413	29.4	(6.8)	101	29.5	(6.7)	312	29.4	(6.8)	0.924
sBG	371	103.6	(34.9)	101	110.8	(41.6)	270	100.9	(31.8)	**0.015**
WBC	409	6	(1.7)	101	5.5	(1.7)	308	6.2	(1.7)	**< 0.001**
ALC	382	1.4	(0.7)	100	1.3	(0.6)	282	1.4	(0.7)	0.265
ANC	380	3.9	(1.4)	100	3.5	(1.5)	280	4.1	(1.3)	**0.001**
NLR	380	3.6	(2.5)	100	3.5	(2.6)	280	3.7	(2.4)	0.594

SD = standard deviation

Age = age in years; BMI = body mass index; sBG = serum blood glucose; WBC = white blood cell; ALC = absolute lymphocyte count; ANC = absolute neutrophil count; NLR = neutrophil lymphocyte ratio; UVA = University of Virginia; DaVA = Veterans Affairs North Texas Health Care System in Dallas. P-Values were computed to compare DaVA and UVA samples using a chi-square test for the percentage of females (% Female) and two-sample t-tests for all other variables. Significant p-values (e.g., Type I error < 0.05) are in **bold**.

### Absolute Lymphocyte Counts (ALC)

**Fig 1:**
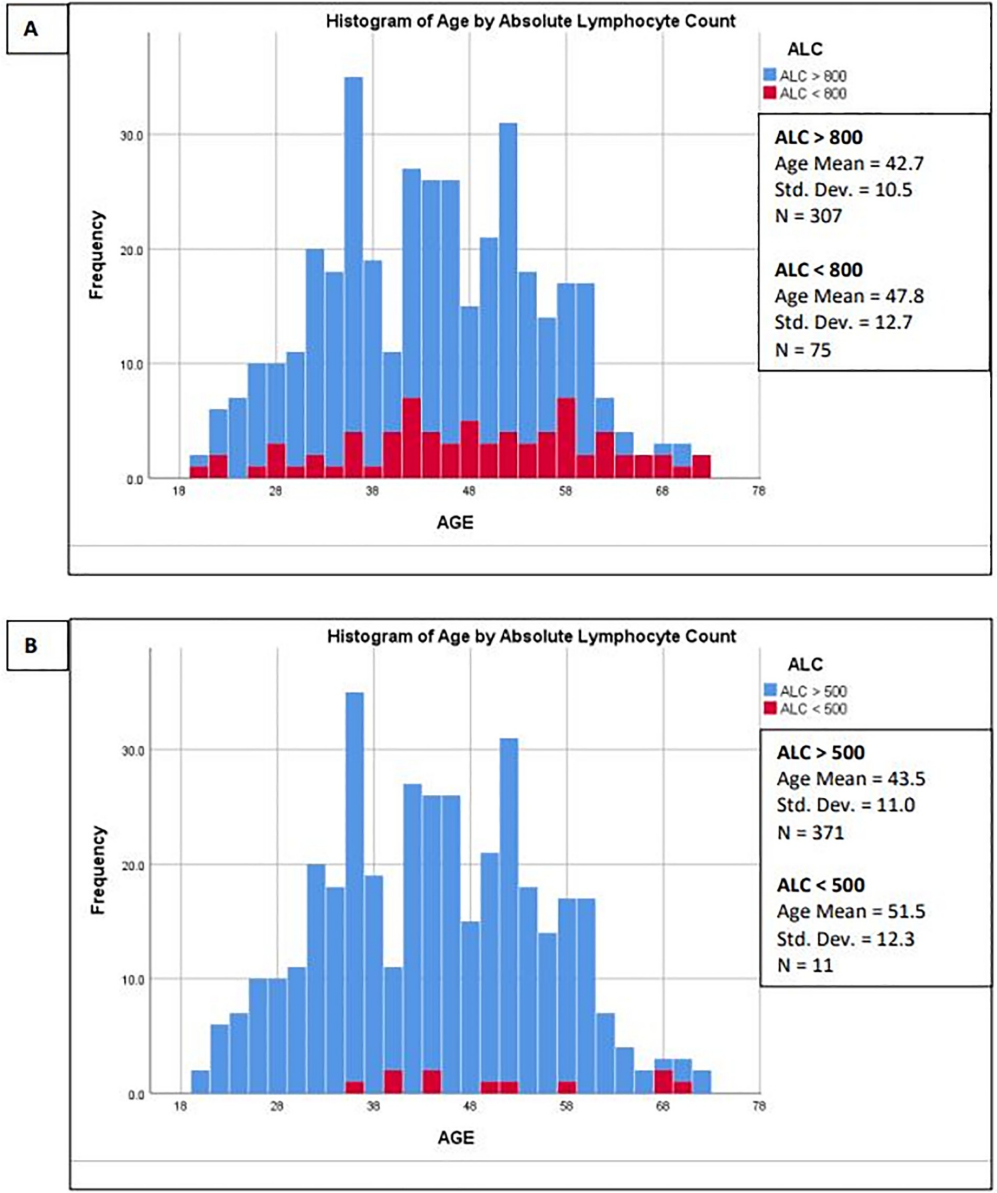
Increasing age is associated with lymphopenia in DMF-treated patients. Figures demonstrates age distribution for (A) absolute lymphocyte count (ALC) > 800. (B) absolute lymphocyte count (ALC) > 500.

On treatment, there was a 30% drop in the mean ALC (Table 1). Nearly 35% of measured ALCs were < 1000 (grade-1 lymphopenia) and 3% were < 500 (grade- 3 lymphopenia). On-treatment, mean ALCs counts were similar between the two centers (Table 1). The incidence of lymphopenia and likelihood for grad-3 lymphopenia increased with age (Fig 1A and 1B). Older individuals were more likely to experience lymphopenia. Grade-3 lymphopenia is only seen in individuals older than 35 years of age and in 33% of patients ≥ 55 years of age (Fig 1A and 1B). Table 2 provides correlations for variables and demonstrates that the ALC was significantly correlated to age UVA & DaVa) and serum blood glucose (DaVA only). In a logistic regression model, age was the greatest predictor of ALC (Table 3). Patient sex, BMI and sBG were not significant predictors of ALC.

**Table 2:** Pearson’s Correlation Coefficient of Serum blood glucose and leukocyte counts (N = 103).

**Combined**	BMI	sBG	NRL	ALC	ANC
Age	0.245	0.128*	0.207*	-0.217*	-0.034
BMI		0.259	0.126	-0.017	0.066
sBG			0.102	-0.027	0.132*
ALC					0.053
**DaVA cohort**	BMI	sBG	NRL	ALC	ANC
Age	0.270	0.550*	0.475*	-0.510*	0.106
BMI		0.381	0.340	-0.050	0.256
sBG			0.445*	-0.284*	0.358*
ALC					-0.051
**UVA cohort**	BMI	sBG	NRL	ALC	ANC
Age	0.232	-0.011	0.122*	-0.159*	-0.126*
BMI		0.317	0.060	-0.061	0.119
sBG			-0.089	0.118	0.037
ALC					0.078

sBG = serum blood glucose; ALC = absolute lymphocyte count; ANC = absolute neutrophil count; NLR = neutrophil lymphocyte ratio

* = p-value < 0.05 after correction for multiple comparisons using Bonferroni method

**Table 3:** Mixed model regression results for ALC < 800, ANC < 1.5, and NLR > 2.0 in the combined cohort (N = 339)*.

Variables	Lymphopenia (ALC < 800)				Neutropenia (ANC < 1.5)				High Neutrophile-Lymphocyte Ratio (NLR > 2.0)			
	Odds Ratio (95% CI)			P-Value	Odds Ratio (95% CI)			P-Value	Odds Ratio (95% CI)			P-Value
Intercept	0.002	(0.00001-	0.198)	0.009	5725.413	(0.092-	3.55e+08)	0.124	0.424	(0.034-	5.259)	0.504
age	1.124	(1.027-	1.229)	0.011	0.943	(0.811-	1.095)	0.44	1.032	(0.986-	1.081)	0.174
female	1.151	(0.226-	5.859)	0.866	0.725	(0.066-	7.948)	0.793	0.492	(0.183-	1.327)	0.161
sBG	1.006	0.990-	1.022)	0.483	0.918	(0.846-	0.996)	0.041	1.003	(0.992-	1.015)	0.558
BMI	0.963	(0.854-	1.087)	0.544	0.974	(0.763-	1.242)	0.829	1.018	(0.947-	1.093)	0.632
UVA	0.339	(0.057-	2.027)	0.236	0.013	(0.0005-	0.346)	0.009	1.979	(0.715-	5.476)	0.189
ANC	0.895	(0.601-	1.334)	0.587								
ALC					0.467	(0.053-	4.132)	0.494				

* sBG = serum blood glucose

ALC = absolute lymphocyte count; ANC = absolute neutrophil count; NLR = neutrophil lymphocyte ratio.

### Absolute Neutrophil Counts (ANC)

There was no notable change in the mean ANC between baseline and on-treatment (Table 1). Comparing cohorts, the mean ANC was lower in the DaVa compared with UVA (3.5 vs 4.1, p = 0.001). On-treatment neutropenia (ANC < 1.5 k/uL), was found 8 individuals (7.8%). Of those who experienced neutropenia while on DMF 63% were male (5 of 8 patients). In those with available sequential lab values were found sustained neutropenia in 4 patients (ANC mean 1.34, range 0.98–1.49) with sequential blood monitoring between 6–12 months from first low ANC value. In the majority of cases, neutropenia occurred in the absence of lymphopenia. Although limited in number, higher sBG values were associated with higher ANC values. Comparing those patients with and without neutropenia, the non-neutropenic (ANC > 1.5) had a significantly higher mean sBG (105± 36 vs 89±16, p-value < 0.0008). ANC was significantly correlated to age (UVA only) and sBG (combined and DaVa). Similarly, those with high sBG (>180) had a higher mean ANC (4.7 ± 1.2 vs 3.9±1.4, p-value = 0.033). In a logistic regression model, sBG was a significant predictor of ANC (Table 3). Age and BMI, were not predictive in the model, nor was lymphocyte count (Table 3). Clinic site was a significant predictor, with neutropenia occurring more often in the DaVa population, which had a higher proportion of males.

### Neutrophil-Lymphocyte Ratio (NLR)

There was a 31% increase in the mean NLR from baseline to on-treatment measures. A NLR cut-off for normal was selected at 2.0 (methods). The mean NLR was higher in those with sBG measured > 180, although this did not reach significance, 3.5 vs. 4.6 (p-value = 0.092), in part due to the small number of high sBG values (>180), found in only 4% of draws. NLR demonstrated significant correlation to age and sBG (Table 2). However, in the logistic regression model, no patient specific factors were predictive of NLR.

## Discussion

Similar to previously published results, we found a ~ 30% reduction in mean ALC in DMF-treated patients, with 3% demonstrating grade-3 lymphopenia. We confirm the findings of Longbrake, et.al., that individuals > 55 yrs of age are at increased risk for grade-3 lymphopenia.^3, 4^ However, we found 33% of patients > 55 yrs demonstrated grade-3 lymphopenia compared to 22% reported in their cohort.^3, 4^ This proportional difference is of uncertain cause, with similar age distribution in both studies (median 43 and 45 years of age). We are the first to report neutropenia with DMF use, which occurred in 7.8% of patients, with 3.9% demonstrating sustained neutropenia. The incidence of incidental neutropenia in a general population is 1.3%, with values reaching up to 4.5% in black individuals [16]. Although we were unable to collect confirmed race classification on our patients in this retrospective study, our results are still higher than what would be expected and suggest these findings to be more than incidental in nature. The main risk factor was sex, with neutropenia being more common in male patients. The inclusion of a veteran population in our study, increased the relative number of males and likely permitted the identification of neutropenia in our results.

A recent paper reported on a single patient with well-controlled Type-1 diabetes, who began to experience glucose dys-regulation after starting DMF that was improved with discontinuation of DMF. In alignment with this report, we found an overall increase in mean sBG across all three groups on treatment (Table 1). In this retrospective cohort, we were only had random non-fasting sBG levels available and had limited number of abnormal values. This limited our ability to conclusively define the relationship between sBG and leukocyte outcomes. Based on contemporary literature, we would expect glucose intolerance (i.e. higher serum blood glucose) to result in higher neutrophils and NLRs, which are the trends found in our data. The NLR was abnormal at baseline in more than 50% of our cohort. A recent paper, reported NLR in MS patients with and without relapse compared to controls^12^. The authors found that the NLR in relapsing patients was 4.89 compared with 2.73 and 2.1 in non-relapsing and controls, respectively. Interestingly, our on-treatment NLR mean was 3.6, between these 2-MS groups in the other study. An elevated NLR has been reported in patients with obesity, diabetes, and metabolic syndrome. In these populations, there is a generalized inflammatory condition that increases circulating neutrophils and the NLR. In addition to being a marker of inflammation, high NLR has been found to have prognostic importance in ischemic heart disease, stroke, and cancer. The clinical importance of the baseline and on-treatment NLR elevation, needs to be further explored in MS. High NLRs may contribute to the worsened outcomes reported in MS patients with co-morbid illness.

### Strengths and limitations

The limitations of this study reflect its retrospective nature. Most patients had their labs drawn as part of routine clinic care, so the timing of draws and subsequent serum blood glucose levels were not regulated. This limited our ability to explore the importance of serum BG on leukocyte outcomes. The interval between a patient’s labs and the type of labs varied based on the physician’s decision for clinical treatment. For that reason, we were not able to obtain every data points at each lab draw. There were some differences between the two center-based populations, specifically differences in age and sex. We believe that this may have enriched our pooled data and permitted deeper assessment of the impact of sex on outcomes.

## Conclusions

This is an exploratory retrospective study of 2-US multiple sclerosis centers of the relationship between patient-specific factors and leukocyte response and ratios DMF-treated patients. DMF-associated lymphopenia occurs in a high proportion of older patients, with grade-3 lymphopenia found in 33% of older patients. We believe this to be the first report of DMF-associated neutropenia in MS patients, which was found in 7.8% of patients following treatment start. Importantly, neutropenia was more common in males and appeared to be independent of lymphopenia. The neutrophil-lymphocyte ratio is abnormal in MS patients and further increased in the setting of DMF-treatment. The clinical importance of this merits further investigation. Our results were not conclusive, but suggest patients with high sBG have elevated neutrophil counts and NLR. Studies in larger patients with glucose intolerance are needed to fully understand these relationships and their potential clinical impact. In summary, patient-specific factors- such as age, sex, and potentially serum BG, appear to modulate leukocyte response and should be integrated into clinical decision making.

## Supporting information

S1 File DMF and Leukocytes_PLOSone_data_DOS. (XLS)

## Abbreviations

ALC: absolute lymphocyte count
ANC: absolute neutrophil count
BMI: body mass index
DaVA: Veterans Affairs North Texas Health Care System in Dallas
DMF: Dimethyl Fumarate
NLR: neutrophil lymphocyte ratio
PML: progressive multifocal leukoencephalopathy
sBG: serum blood glucose
UVA: University of Virginia
WBC: white blood cell
